# Effectiveness of General Practitioner Referral Versus Self-Referral Pathways to Guided Internet-Delivered Cognitive Behavioral Therapy for Depression, Panic Disorder, and Social Anxiety Disorder: Naturalistic Study

**DOI:** 10.2196/68165

**Published:** 2025-03-25

**Authors:** Jill Bjarke, Rolf Gjestad, Tine Nordgreen

**Affiliations:** 1 Division of Psychiatry, Haukeland University Hospital, Haukelandsbakken 2, Bergen, 5009, Norway, 47 93057985; 2 Department of Global Public Health and Primary Care, University of Bergen, Bergen, Norway; 3 Centre for Research and Education in Forensic Psychiatry, Haukeland University Hospital, Bergen, Norway

**Keywords:** referral pathway, GP-referral, self-referral, guided internet-delivered cognitive behavioral therapy, ICBT, routine care clinic, depression, panic disorder, social anxiety disorder, psychological therapy, referrals, cognitive, behavioral therapy, anxiety, SAD, treatment effectiveness, mental health, pathways

## Abstract

**Background:**

Therapist-guided, internet-delivered cognitive behavioral therapy (guided ICBT) appears to be efficacious for depression, panic disorder (PD), and social anxiety disorder (SAD) in routine care clinical settings. However, implementation of guided ICBT in specialist mental health services is limited partly due to low referral rates from general practitioners (GP), which may stem from lack of awareness, limited knowledge of its effectiveness, or negative attitudes toward the treatment format. In response, self-referral systems were introduced in mental health care about a decade ago to improve access to care, yet little is known about how referral pathways may affect treatment outcomes in guided ICBT.

**Objective:**

This study aims to compare the overall treatment effectiveness of GP referral and self-referral to guided ICBT for patients with depression, PD, or SAD in a specialized routine care clinic. This study also explores if the treatment effectiveness varies between referral pathways and the respective diagnoses.

**Methods:**

This naturalistic open effectiveness study compares treatment outcomes from pretreatment to posttreatment and from pretreatment to 6-month follow-up across 2 referral pathways. All patients underwent module-based guided ICBT lasting up to 14 weeks. The modules covered psychoeducation, working with negative or automatic thoughts, exposure training, and relapse prevention. Patients received weekly therapist guidance through asynchronous messaging, with therapists spending an average of 10‐30 minutes per patient per week. Patients self-reported symptoms before, during, immediately after, and 6 months posttreatment. Level and change in symptom severity were measured across all diagnoses.

**Results:**

In total, 460 patients met the inclusion criteria, of which 305 were GP-referred (“GP” group) and 155 were self-referred (“self” group). Across the total sample, about 60% were female, and patients had a mean age of 32 years and average duration of disorder of 10 years. We found no significant differences in pretreatment symptom levels between referral pathways and across the diagnoses. Estimated effect sizes based on linear mixed modeling showed large improvements from pretreatment to posttreatment and from pretreatment to follow-up across all diagnoses, with statistically significant differences between referral pathways (GP: 0.97‐1.22 vs self: 1.34‐1.58, *P*<.001-.002) and for the diagnoses separately: depression (GP: 0.86‐1.26, self: 1.97‐2.07, *P*<.001-.02), PD (GP: 1.32‐1.60 vs self: 1.64‐2.08, *P*=.06-.02) and SAD (GP: 0.80‐0.99 vs self: 0.99‐1.19, *P=*.18-.22).

**Conclusions:**

Self-referral to guided ICBT for depression and PD appears to yield greater treatment outcomes compared to GP referrals. We found no difference in outcome between referral pathway for SAD. This study underscores the potential of self-referral pathways to enhance access to evidence-based psychological treatment, improve treatment outcomes, and promote sustained engagement in specialist mental health services. Future studies should examine the effect of the self-referral pathway when it is implemented on a larger scale.

## Introduction

### Background

Depression and anxiety disorders are recognized as major contributors to global disability, carrying significant societal costs and having high personal impact [1]. In 2019, nearly 600 million people worldwide were affected by these conditions. Throughout life, depression and anxiety disorders are approximately 50% more common in women than in men [2]. Broadly accessible treatment is required to reduce this burden [3], yet a significant treatment gap remains between the need for and access to adequate care [4]. This gap is driven by a variety of factors, including limitations in available health care services, financial barriers, avoidance of help-seeking, lack of mental health literacy, and stigma [5]. The dominant model of treatment delivery—face-to-face treatment with trained mental health professionals in clinical settings—further restricts the widespread dissemination of mental health care [6]. Even in high-income countries, where access to care is more readily available, only about one-third of those with major depressive disorders receive formal mental health care [2].

Pharmacological and psychological therapies have demonstrated equal effects in treating depression and anxiety disorders [7,8]. However, psychological therapy is often preferred by patients over medication due to having fewer side effects and better long-term outcomes [9,10]. Cognitive behavioral therapy (CBT) is the psychological treatment with the strongest empirical support [11] and it is the recommended first-line treatment for these disorders [12,13]. Internet-delivered cognitive behavioral therapy (ICBT) delivers evidence-based CBT specifically targeting, but not limited to, depression and anxiety disorders [14]. ICBT offers several practical advantages that help address the treatment gap, including reduced travel time and expenses, greater flexibility to fit around individuals’ daily schedules, and the potential to overcome stigma-related barriers through increased anonymity [2,15]. Additionally, the internet-delivered treatment format is less time-consuming for the therapist, thus it is scalable and affordable without compromising the quality of care [16]. These factors make ICBT an attractive option for expanding access to mental health treatment, particularly in areas with limited resources or where traditional face-to-face therapy is not readily available.

Systematic reviews have found the effect of guided ICBT for depression and anxiety disorders to be no different from that of face-to-face CBT [17-19]. Guided ICBT for depression and anxiety is found to work well in routine care clinics and tends to replicate results found in efficacy studies in Sweden, Denmark, Norway, Canada, and Australia [20-24] and to have long-term effects [25]. However, the implementation of guided ICBT in specialist mental health care has been slow, partly due to lack of knowledge, prejudice, and negative attitudes among health care professionals and general practitioners (GPs) [26]. This is concerning, as GPs in primary care often serve as gatekeepers and are responsible for initiating referrals to secondary care and specialist clinics.

The lack of referral from GPs to guided ICBT [26] has led to efforts enhancing access to care, with self-referral being proposed as a way to improve access to psychological therapies [27]. Self-referral implies that patients can seek the service from secondary care or specialist clinics, bypassing the need for referrals from GPs [27]. Self-referral to ICBT opens a pathway to evidence-based psychological care especially for individuals who never reach specialist clinics or mention their problems when consulting their GP [15]. Some have suggested that self-referral may attract more motivated patients [11], which could influence treatment engagement and improve outcomes [28]. Participants in guided ICBT trials tend to be more educated [15]. Since higher education is associated with better health literacy and access to and understanding of health information [29,30], patients self-referred to ICBT may thus be more responsive to treatment, which could help explain potential differences in treatment outcomes.

In addition, self-referral pathways are believed to empower patients by giving them greater control over their health care [31]. According to Self-Determination Theory (SDT), having greater control over the decision to seek care may foster autonomy, which in turn could enhance motivation and engagement with treatment [32]. For example, patients who self-refer may feel more motivated, ready, and confident in their choice to pursue ICBT, which can lead to greater engagement in therapy. When autonomy is supported by a sense of competence, such as patients feeling capable of managing their treatment, it can strengthen intrinsic motivation. This, combined with supportive feedback from ICBT therapists and a sense of relatedness, can also strengthen intrinsic motivation and may thus encourage continued participation and adherence to treatment plans [33].

However, while self-referral can improve access to psychological therapies, it also presents challenges. One concern is that self-assessment tools for depression and anxiety are not validated for lay self-diagnosis in this context. This is particularly important when accurate diagnosis is crucial for providing appropriate evidence-based treatment [34]. It has been argued that self-referral in general may result in unnecessary, costly, or even harmful interventions. Conversely, it could also lead to reduced patient responsibility, causing symptoms to be dismissed or action to be delayed, potentially leading to harm [35]. This reliance on individual health care–seeking behaviors has been identified as a factor that can contribute to widening socioeconomic inequalities [36]. Additionally, self-referral may lead to oversaturation of specialist health care and potentially widen already existing health inequalities by primarily attracting younger, well-educated woman. However, this concern remains underexplored and may be context-dependent [36].

The practice of using self-referral pathways to specialist care varies across countries and clinical domains, with physiotherapy and mental health services being among the most common [37]. Self-referral is well studied in the field of physiotherapy and is an available pathway to musculoskeletal care in many countries [38]. Consistent yet limited evidence suggests that self-referral for musculoskeletal care yields clinical outcomes comparable to GP referrals [39]. Research comparing different referral pathways to mental health services remains limited. In a recent systematic review examining who benefits from guided internet-based interventions across mental health diagnoses, 88 predictors and moderators of treatment outcome were analyzed but referral pathway to treatment was not included [40]. A recent study recommends investigating referral pathways on patient outcomes [41]. However, some studies comparing referral pathways to psychological care already exist.

First, in a study on GP referral and self-referral to psychological treatment for patients with severe health anxiety, Hoffmann et al [11] examined the accuracy of these referral pathways in recruiting patients with treatment-demanding symptom levels. The accuracy was assessed by comparing the proportion of patients in each referral group who met the treatment criteria, with results significantly favoring self-referral. One reason for this difference was that several GP-referred patients did not attend the clinical diagnostic interview and therefore were excluded from the study. The findings suggest that self-referral may be a more accurate method for recruiting patients with severe health anxiety, as self-referred patients not only meet the criteria for treatment but also appear to be more motivated to participate in it [11].

Referral pathway has also been studied in relation to how consistently patients attend psychological therapy sessions within Improving Access to Psychological Therapies (IAPT) services [42]. When comparing GP referral, GP-initiated self-referral, and true self-referral to IAPT, no significant differences were found between referral pathways and attendance at the subsequent therapy sessions. Moreover, the study examined the patient’s preferred pathway and found that those who had a GP-initiated self-referral later stated a preference for the GP to take full responsibility for the referral process. Accordingly, 60% of the true self-referrers stated that they preferred to self-refer again if they needed additional services from IAPT [42].

Although studies on treatment outcomes across referral pathways to psychological therapy are scarce, a notable exception is an observational study comparing GP referral and self-referral to 2 similar ICBT treatments for depression and/or anxiety [43]. In this study, patients from both referral pathways reported significant symptom reduction; however, those who self-referred showed larger effect sizes both at posttreatment and at the 3-month follow-up compared to those referred by their GPs [43].

No studies have yet investigated the role of referral pathway on treatment outcomes for guided ICBT for depression and anxiety disorders to a specialized routine care clinic. Based on the results from the comparison of GP referral and self-referral pathways to ICBT [11,43], we hypothesize that individuals who self-refer to specialized mental health care services will experience greater treatment effectiveness from pretreatment to posttreatment and for pretreatment to 6-month follow-up compared to those referred by GPs. Additionally, we will explore differences in treatment effectiveness across the specific diagnoses in relation to referral pathways.

### Aim

The aim of this study was to compare the overall treatment effectiveness across different referral pathways—GP-referred and self-referred—in guided ICBT for moderate depression, panic disorder (PD), and social anxiety disorder (SAD). We also explore whether differences in treatment effectiveness between the referral pathways vary across the 3 diagnoses.

## Methods

### Ethical Considerations

This study was approved by the Regional Committee for Medical Research Ethics (REK) 2014/2175. The authors assert that all procedures contributing to this work comply with the ethical standards of the relevant national and institutional committees on human experimentation as well as with the principles of the Declaration of Helsinki [44]. The original written informed consent covers secondary analysis without additional consent. Data were deidentified, with direct personal identifiers removed and the key linking IDs stored separately on an inaccessible server. No compensation was provided for participation.

### Setting

The data collection for this study was conducted at the eCoping clinic, a specialized routine mental health care clinic at Haukeland University Hospital in Bergen, Norway. All patients were referred to the eCoping clinic either from their GP or by themselves through direct contact with the eCoping team, resulting in both GP referrals and self-referrals. This study presents data from GP-referred patients included between September 2014 and May 2019 and self-referred patients included between September 2016 and May 2019.

### Design

This study was a naturalistic open effectiveness study with repeated assessments for primary treatment outcomes and a 6-month follow-up for patients with moderate depression, PD, and SAD undergoing therapist-guided ICBT.

### Referral to Treatment

Several approaches were used to increase knowledge about eCoping among the GPs. First, there were face-to-face educational visits conducted by the eCoping team. Second, GPs were provided with test-user accounts for the eCoping program to familiarize themselves with the treatment, however, none of the GPs logged in. Third, information about the treatment was presented in GPs’ waiting rooms through flyers and short messages on information screens. In addition, promotion of eCoping was carried out through the local newspapers to highlight the new referral option. Finally, for a short period, Facebook ads about the possibility to self-refer were targeted at the local population.

GPs evaluated patients using their clinical judgment to assess symptom severity to determine the need for specialized care services; if deemed necessary, the GP authored a referral to the eCoping clinic. Patients who self-referred sent an email with their contact details to an address available on the eCoping website [45]. Subsequently, an eCoping therapist conducted a clinical interview by telephone to assess symptom severity and the treatment’s relevance. A summary of the interview was generated as a self-referral.

A specialist in clinical psychology reviewed all referrals regardless of referral pathway in accordance with national priority guidelines [46] to determine eligibility for specialized care treatment.

As the study was conducted within routine care, the eligibility assessment and the inclusion and exclusion criteria were identical across referral pathways, ensuring that the sample was unbiased with respect to referral source. Inclusion criteria for all study patients were: (1) being 18 years of age or older; (2) diagnosed either with major depressive episode, SAD, or PD; (3) if using antidepressants, being on a stable dosage over the previous four weeks; and (4) fluent in oral and written Norwegian. Exclusion criteria for all study patients were: (1) current suicidal ideation, (2) current psychosis, (3) current substance abuse, (4) using benzodiazepines daily, (5) immediate need of other treatment, and (6) no access to the internet. Written informed consent was obtained from all study patients prior to data collection. We have no data on individuals who were screened out during the eligibility process as these patients did not sign informed consent forms. Patients who met the criteria for treatment received a scheduled appointment for a face-to-face consultation.

### Procedure

During the face-to-face consultation, all patients underwent a diagnostic interview with the Mini-International Neuropsychiatric Interview (MINI) [47]. Based on the MINI, patients deemed unfit for eCoping were excluded and rereferred to a more suitable treatment option. The treatment program allocation was determined based on the MINI assessment.

### Training

All therapists at the eCoping clinic were colocated for 1-2 days per week when working with guided ICBT, with an ordinary workload during the rest of the week. In addition to a 1-year continuing education program, the therapists received weekly peer supervision and monthly expert supervision from the Internet Psychiatry Clinic in Stockholm.

### Treatment

For depression, the guided ICBT program included 8 text-based modules including psychoeducation, behavioral activation, and cognitive reappraisal and relapse prevention. PD was addressed with 9 text-based modules with psychoeducation, working with automatic thoughts, behavioral experiments, in vivo exposure, and relapse prevention. Similarly, SAD treatment comprised 9 text-based modules including psychoeducation, working with automatic thoughts, behavioral experiments, shifting focus, and relapse prevention. The treatments are described in detail in previous publications [22,48,49]. The therapists adhered to the treatment protocol and provided uniform treatment to all patients regardless of referral pathway. The treatment programs were provided on a secure web platform that was state-of-the-art when data collection started in 2014.

Treatment time for the 3 diagnoses was up to 14 weeks. Irrespective of treatment program, each patient was expected to spend 7‐10 days per module; access to the next module was gained upon finishing the previous one. Each module required approximately 45 minutes to complete.

After each completed module or at least once per week, a therapist gave feedback and guidance tailored to individual patient needs based on their worksheets, symptom assessment, and emails, while also introducing them to the next module. All feedback and communication were enabled asynchronously through a secure email system. Therapists spent an average of 10‐30 minutes per patient per week. Patients not heard from for 1 week were contacted by the therapist via an SMS text message to encourage them to continue to work through the program. When necessary, phone calls could be made to solve problems, discuss motivation, or simply get in touch with an inactive patient.

### Primary Outcomes

All self-report measures and questionnaires were administered via the internet and made accessible at the end of each module. Patients completed the measures and questionnaires pretreatment, after each module, posttreatment, and at the 6-month follow-up. The programs for depression, PD, and SAD had the following primary outcome measures:

- Depression: Montgomery Åsberg Depression Rating Scale, Self-rating version (MADRS-S) [50]. The MADRS-S comprises 9 items rated on a Likert scale from 0‐6 (total score range: 0‐54), where higher scores indicate more severe depression. The scale has been found to be sensitive to change [50,51] and has shown high correlations between expert ratings and self-reports [50]. Internal consistency measured with Cronbach α yielded 0.77 for patients with GP referral and 0.82 for self-referred patients.
- Panic disorder: Body Sensation Questionnaire (BSQ) [52]. The BSQ has been found sensitive to symptom change during treatment [52]. The BSQ comprises 16 items rated on a 5-point Likert scale (total score range: 16‐80), where higher scores indicate a higher level of fear and sensitivity to bodily sensations commonly experienced during autonomic nervous system arousal. Cronbach α yielded 0.84 for GP-referred patients and 0.88 for self-referred patients and showed good internal consistency reliability.
- Social anxiety disorder: Social Phobia Scale (SPS) [53]. The SPS measures social phobia and the distress of being observed or watched while performing daily activities in the presence of others [53]. The SPS entails 20 questions rated on a 5-point Likert scale (total score range: 0‐100), where higher scores indicate higher anxiety of being observed or scrutinized. The scale has shown good reliability and validity [53,54], as well as discriminant validity in distinguishing individuals diagnosed with SAD from both healthy controls and individuals with other anxiety disorders [53]. Internal consistency measured with Cronbach α yielded 0.91 for GP-referred patients and 0.93 for self-referred patients.

To address the main aim of this study, we combined the outcome measures for the 3 treatment modalities. We harmonized the outcome total scores and computed a common harmonized outcome measure (see Equation 1), thereby increasing the net sample and the statistical power for the analyses [55]. To ensure comparability across different measurements, this formula normalizes the total score by first subtracting the minimum possible value, then dividing by the range (maximum possible value minus minimum), and finally multiplying by 100. This transformation ensures that all scores are expressed on a common scale, reducing biases introduced by different measurement units. However, the harmonization removes the original scale’s absolute meaning and assumes that all scales represent the same underlying construct in a comparable way, which may not always be true if the scales have different distributions or nonlinear relationships. Moreover, harmonization was only feasible at the total score level. At the item level, where variables were ordinal, no harmonization was possible since no patients could have information on all 3 outcome variables. Therefore, we were unable to calculate internal consistency for the harmonized outcome measure.


(1) $$H=\;\frac{score-MIN}{MAX-MIN}*100$$

### Statistics

Data preparation and calculation of descriptive statistics and bivariate analyses, including percentages, means, standard deviations, and cross tabulations with *χ*^2^ tests, were conducted using IBM SPSS (version 29; IBM Corp) [56]. Effect sizes from pretreatment to posttreatment and from pretreatment to 6-month follow-up are reported as Cohen *d*, based on pooled standard deviations [57]. All measurement points (completed modules) were used for the analyses; however, the focus is the between-group (GP-referred vs self-referred) difference in pretreatment levels and changes from pretreatment to posttreatment and from pretreatment to 6-month follow-up. We performed analyses of the treatment outcome using linear mixed modeling (LMM). LMM is a recommended statistical method for handling missing data under the assumption of missing at random and uses all available data for estimation [58]. We analyzed levels and changes in data with a random intercept and fixed slope model. First, unconditional models including the time variable were tested. Time was defined as modules, giving changes in outcomes per module. To compare group differences over time, we added the referral group, both as a main effect and in an interaction effect with time. The reliable change index (RCI) was calculated using individual-level changes from pretreatment to posttreatment and from pretreatment to the 6-month follow-up, based on observed data for patients in each referral pathway and diagnosis. The RCI calculates whether changes in symptoms are reliable and not caused by measurement error [59]. Symptom level was considered to have improved if the outcome measure indicated a reliable change, as defined by the RCI [59]. The RCI was calculated with the formula 1.96 × SD × √2(1-Rel), where SD is the observed standard deviation and Rel is the internal consistency at pretreatment assessment for each referral pathway and outcome measure. Improvement was defined by a negative RCI change, while deterioration was defined by a positive RCI change. Sensitivity models based on multiple imputation (MI) were analyzed to explore possible differences in levels and changes related to missing data for the 2 groups, assuming missing data were missing at random [60]. Pretreatment and longitudinal information were used as predictors of plausible values, and 50 data sets were generated. To account for the clustered data structure in the imputation process, data were transformed into wide format and analyzed with latent growth curve models. These models were parameterized identically to the corresponding linear mixed effect models, ensuring the same number of parameters and constraints [61]. Imputations and analyses were conducted using Mplus 8.10 [62].

## Results

### Patients

A total of 460 patients provided informed consent across the 2 referral pathways. Patient characteristics are shown in Table 1. Of the total sample, approximately two-thirds were referred by a GP (GP-referred), while about one-third were self-referred. There were more females in both groups (GP-referred: 176/289, 60.9%; self-referred: 106/151, 70.2%). The mean age across the total sample was approximately 32 years, with an average duration of complaints of about 10 years in both groups. The only statistically significant difference between the 2 groups was that a higher proportion of those who self-referred had obtained a university-level education. The distribution across the diagnosis-specific treatment programs was approximately 22% depression, 38% PD, and 41% SAD. Among patients in the depression group, the referral pathway was approximately equally distributed. In contrast, the pathway distribution for both the PD group and the SAD group was about two-thirds GP referrals and one-third self-referrals.

**Table 1.** Pretreatment characteristics.

Demographics	Total group (N=460^a^)	Self-referred (n=155)	GP-referred^b^ (n=305)	*P* value
**Gender, n** ^**c**^ **/N (%)**				.054
Female	282/440 (64.1)	106/151 (70.2)	176/289 (60.9)	
Male	158/440 (35.9)	45/151 (29.8)	113/289 (39.1)	
**Age (years), n/N, mean (SD)**	460/460, 32.5 (11.0)	155/155, 31.9 (10.3)	305/305, 32.7 (11.4)	.12
**Relationship status, n/N (%)**				.32
Married/cohabitant	225/435 (51.7)	83/151 (55)	142/284 (50)	
Single	210/435 (48.3)	68/151 (45)	142/284 (50)	
**Education, n/N (%)**				<.001
Primary level	60/439 (13.7)	14/151 (9.3)	46/288 (16)	
Secondary level	193/439 (44)	50/151 (33.1)	143/288 (49.7)	
Tertiary level	186/439 (42.4)	87/151 (57.6)	99/288 (34.4)	
**Years with complaints, n/N mean (SD)**	426/460 10.2 (9.5)	147/151, 9.80 (9.43)	279/305, 10.35 (9.62)	.54
**Treatment program, n (%)**				
Depression	101 (21.7)	48 (31)	53 (17.4)	
Panic disorder	172 (37.4)	56 (36.1)	116 (38)	
Social anxiety disorder	187 (40.7)	51 (32.9)	136 (44.6)	

a N: number of patients.

b GP-referred: referred by general practitioners.

c n: number of patients in that subgroup.

### Attrition and Adherence

In the depression group (N=101), 97 patients (96%) completed the MADRS-S assessment pretreatment, 66 (65.3%) completed it at posttreatment, and 41 (40.6%) completed it at the 6-month follow-up. The amount of missing data was found to be equal between the 2 groups (GP-referred: mean 4.9, SD 2.9; self-referred: mean 4.6, SD 3.3; *t*_99_=0.50, *P*=.62). In the PD group (N=172), 156 patients (90.7%) completed the BSQ pretreatment, with 111 (64.5%) and 67 (38.9%) completing the assessment at posttreatment and follow-up, respectively. No difference in the amount of missing data was found between the 2 groups (GP-referred: mean 5.0, SD 3.5; self-referred: mean 3.9, SD 3.1; *t*_170_=2.0, *P*=.051). For the SAD group (N=187), 177 patients (95.2%) completed the SPS pretreatment, followed by 99 (52.9%) at posttreatment and 59 (31.6%) at follow-up, with no difference in the amount of missing data between the groups (GP-referred: mean 5.7, SD 3.6; self-referred: mean 4.9, SD 3.8; *t*_185_=1.50, *P*=.14). Details on the observed diagnosis-specific outcome measures are provided in Table S1 in Multimedia Appendix 1.

### Primary Outcomes

LMM results showed that, when harmonizing the outcome measures for all 3 diagnoses, significant symptom reduction was evident for both referral pathways from pretreatment to posttreatment and pretreatment to 6-month follow-up. Overall, patients who self-referred demonstrated significantly greater estimated symptom reduction from pretreatment to posttreatment and pretreatment to 6-month follow-up compared to those referred by GPs. The estimated scores from the LMM showed that the MADRS-S level decreased over time (Table 2). Self-referred patients showed no significant difference in depression scores from GP-referred patients at the pretreatment assessment, but a statistically greater reduction from pretreatment to posttreatment and pretreatment to 6-month follow-up. More details on the level and change in the estimated outcome measures are provided in Table S2 in Multimedia Appendix 2. Table 3 shows estimated means at pretreatment, posttreatment, and follow-up together with estimated effect sizes. Overall, we found large effect sizes (>0.8) over time.

**Figure 1.**
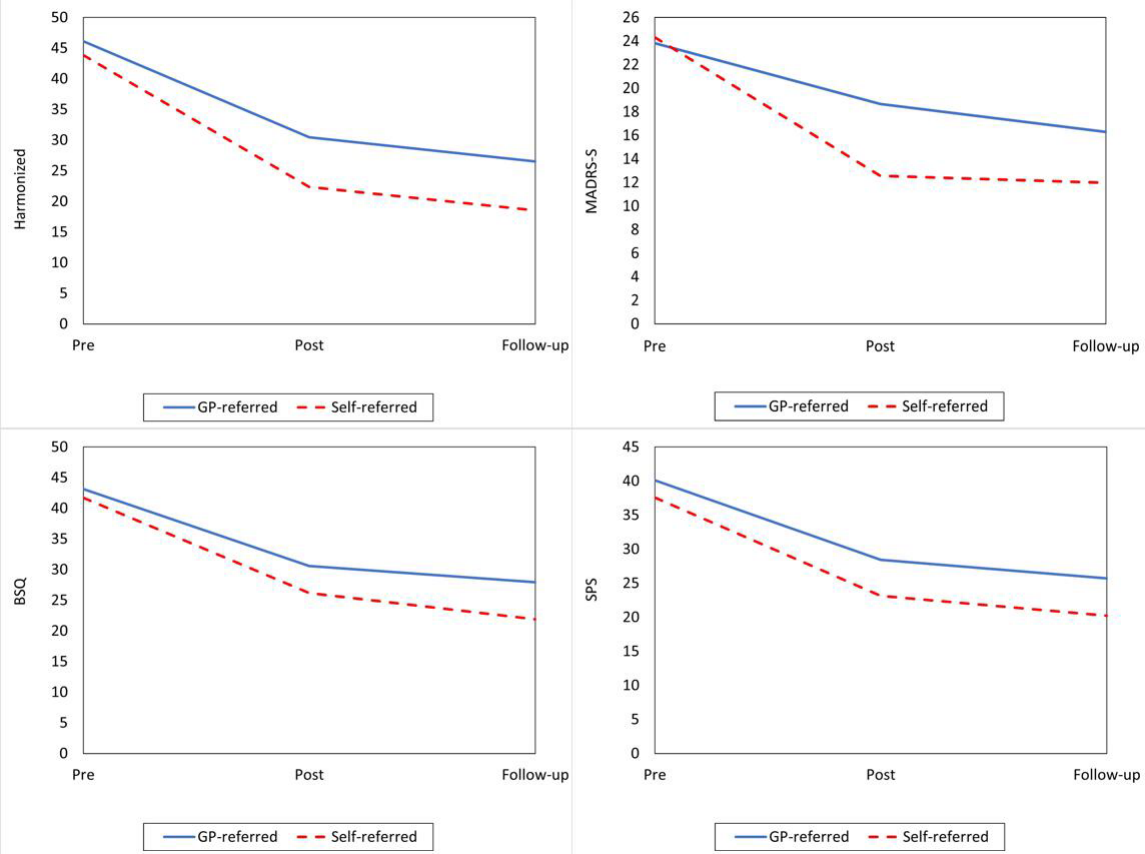
Estimated outcome scores pretreatment, posttreatment, and at 6-month follow-up. BSQ: Body Sensation Questionnaire; follow-up: 6-month follow-up; GP: general practitioner; MADRS-S: Montgomery Åsberg Depression Rating Scale, Self-rating version; pre: pretreatment; post: posttreatment; SPS: Social Phobia Scale.

Figure 1 depicts the corresponding level and change in the LMM. Overall, the figure shows that the estimated harmonized levels for both referral pathways decreased over time, with statistically significant greater reductions from pretreatment to posttreatment and from pretreatment to 6-month follow-up for those who self-referred. The pattern of changes in MADRS-S showed a somewhat different picture compared to the other results, with a stronger reduction in the self-referred group in the pretreatment to posttreatment interval, but no further reduction from posttreatment to follow-up. BSQ levels for both referral pathways decreased over time. Self-referred patients showed a significantly greater reduction in BSQ scores from pretreatment to 6-month follow-up and a temporarily greater reduction after completing module 2. The LMM scores showed that the estimated SPS levels and changes did not differ between the referral pathways from pretreatment to 6-month follow-up. Self-referred patients showed a temporary significantly greater reduction from pretreatment to module 3.

**Table 2.** Estimated outcome measures over time for general practitioner–referred and self-referred groups.

	Harmonized outcome		MADRS-S^a^		BSQ^b^		SPS^c^	
	*b*	*P* value	*b*	*P* value	*b*	*P* value	*b*	*P* value
Pre^d^	46.13	<.001	23.82	<.001	43.15	<.001	40.12	<.001
Post^e^	−15.67	<.001	−5.15	<.001	−12.57	<.001	−11.68	<.001
Follow-up^f^	−19.63	<.001	−7.54	<.001	−15.21	<.001	−14.40	<.001
**Group differences**								
Self-referred^g^	−2.26	.23	0.50	.76	−1.42	.44	−2.55	.35
Self-referred × post	−5.85	<.001	−6.60	<.001	−3.00	.06	−2.75	.18
Self-referred × follow-up	−5.72	.002	−4.80	.02	−4.62	.02	−2.93	.22

a MADRS-S: Montgomery Åsberg Depression Rating Scale, Self-rating version.

b BSQ: Body Sensation Questionnaire.

c SPS: Social Phobia Scale.

d Pre: pretreatment.

e Post: posttreatment.

f Follow-up: 6-month follow-up.

g Reference group: general practitioner–referred.

**Table 3.** Estimated outcome measures pretreatment, posttreatment, and at follow-up.

		Pre^a^	Post^b^		Follow-up^c^	
		Mean^d^	Mean	Effect size	Mean	Effect size
**Harmonized outcome** ^**e**^						
	GP^f^	46.13	30.46	−0.97	26.50	−1.22
	Self^g^	43.87	22.35	−1.34	18.52	−1.58
**MADRS-S** ^**h**^						
	GP	23.82	18.67	−0.86	16.28	−1.26
	Self	24.32	12.57	−1.97	11.98	−2.07
**BSQ** ^**i**^						
	GP	43.15	30.58	−1.32	27.94	−1.60
	Self	41.73	26.16	−1.64	21.90	−2.08
**SPS** ^**j**^						
	GP	40.12	28.44	−0.80	25.72	−0.99
	Self	37.57	23.14	−0.99	20.24	−1.19

a Pre: pretreatment.

b Post: posttreatment.

c Follow-up: 6 months follow-up.

d Mean: model-estimated mean values.

e Harmonized outcome: harmonization of MADRS-S, BSQ, and SPS.

f GP: GP-referred.

g Self: self-referred.

h MADRS-S: Montgomery Åsberg Depression Rating Scale, Self-rating version.

i BSQ: Body Sensation Questionnaire.

j SPS: Social Phobia Scale.

### Reliable Change

Overall, of those who self-referred, statistically significantly more patients showed an improvement in reliable change index (RCI) from pretreatment to posttreatment compared to those referred by their GP (Table 4). From pretreatment to follow-up, we found statistically significant differences only for self-referred patients with PD. RCI improvement based on the observed data required a reduction of at least 8 and 9 points on the MADRS-S (GP and self-referred), 12 and 11 points on the BSQ (GP and self-referred), and 13 points on the SPS (both referral pathways). There were statistically significant differences in RCI improvement between referral pathways among patients with depression from pretreatment to posttreatment, as well as among patients with PD from pretreatment to posttreatment and from pretreatment to follow-up. In all cases, symptom improvement favored those who self-referred. Of patients with depression or PD who reported no change in symptom level at posttreatment and at the 6-month follow-up, the majority were GP-referred. In contrast, the reliable change in reported symptom level between the referral pathway among patients with SAD was minor at both posttreatment and the 6-month follow-up. The proportions of patients with symptom improvement and no change in SPS were evenly distributed between the referral pathways.

**Table 4.** Reliable change in outcome measures.

	Total^a^		MADRS-S^b^		BSQ^c^		SPS^d^	
	GP^e^, n (%)	Self^f^, n (%)	GP, n (%)	Self, n (%)	GP, n (%)	Self, n (%)	GP, n (%)	Self, n (%)
**Posttreatment**								
Improved	81 (44.8)	64 (64)	9 (27.3)	21 (70)	37 (51.4)	27 (71.1)	35 (46.1)	16 (50)
No change	94 (51.9)	36 (36)	21 (63.6)	9 (30)	35 (48.6)	11 (28.9)	38 (50)	16 (50)
Deterioration	6 (3.3)	0 (0)	3 (9.1)	0 (0)	0 (0)	0 (0)	3 (3.9)	0 (0)
*P* value^g^		<.001		.002		.047		.51
**Follow-up** ^**h**^								
Improved	56 (57.7)	50 (72.5)	9 (45)	14 (66.7)	28 (66.7)	22 (91.7)	19 (54.3)	14 (58.3)
No change	40 (41.2)	19 (27.5)	11 (55)	7 (33.3)	14 (33.3)	2 (8.3)	15 (42.9)	10 (41.7)
Deterioration	1 (1)	0 (0)	0 (0)	0 (0)	0 (0)	0 (0)	1 (2.9)	0 (0)
*P* value		.04		.16		.02		.69

a Total: Sum of improved, sum of no change, and sum of deterioration from MADRS-S, BSQ, and SPS.

b MADRS-S: Montgomery Åsberg Depression Rating Scale, Self-rating version.

c BSQ: Body Sensation Questionnaire.

d SPS: Social Phobia Scale.

e GP: GP-referred.

f Self: self-referred.

g Chi-square test for differences in distributions for improved, no change, and deterioration between referral pathways.

h Follow-up: 6-month follow-up.

### Missing Data Sensitivity Analyses

The sensitivity analyses using MI indicated that the estimated pretreatment MADRS-S mean score for the GP-referred group was identical to the full-information maximum likelihood (FIML) estimate (Multimedia Appendix 3) . However, the reduction in symptoms from pretreatment to 6-month follow-up was smaller in the MI analysis compared to the original analysis (FIML=–7.54 vs MI=–6.55). Additionally, we also found a difference in the group effect of self-referral at the 6-month follow-up (FIML=–4.80 vs MI=5.49). This suggests less reduction and less difference in reduction in depression symptom levels in the net population based on the sample with the observed information compared to the total population with intact information on all variables and all measurement points. Thus, the main results may have shown somewhat overestimated reductions. A similar pattern was found for BSQ (GP follow-up levels: FIML=–15.21 vs MI=–14.21). For SPS, the difference was smaller but in the opposite direction (GP follow-up levels: FIML=–14.40 vs MI=–15.26). Differences from pretreatment to posttreatment are also reported in Multimedia Appendix 3; however, since there were fewer missing data at this assessment point, the results closely matched those of the main analysis.

## Discussion

This study compared the overall treatment effectiveness of guided ICBT across GP-referred and self-referred pathways for patients with moderate depression, PD, or SAD. We also explored whether differences in treatment effectiveness between the referral pathways varied across the diagnoses separately. All patients underwent guided ICBT in a specialized routine mental health care clinic.

### Principal Findings

Overall, there were large effect sizes of guided ICBT from pretreatment to posttreatment and from pretreatment to 6-month follow-up, aligning with the large effects reported in a systematic review of routine care practice on the effectiveness of guided ICBT for depression and anxiety [63], and further aligning with treatment results from previous investigations from the same specialized routine care clinic [22,48,49].

Our overall results support the hypothesis that patients who self-refer have significantly larger treatment effectiveness compared to those referred by their GP. This is evident from the relatively large estimated effect sizes in the harmonized scores in the self-referred group (effect sizes: 1.34‐1.58) compared to the GP-referred group (effect sizes: 0.97‐1.22). Additionally, there was a significant difference in reliable change between the self-referred and GP-referred groups. These results are noteworthy, especially since no difference in pretreatment symptom level was found across referral pathways. Our results favoring self-referral are consistent with those of Staples et al [43] where self-referred patients showed greater effect sizes compared to GP-referred patients both from pretreatment to posttreatment and from pretreatment to the 3-month follow-up. Neither our study nor previous studies on referral pathway to ICBT [43] investigated the potential mechanisms behind the findings that self-referred patients have better treatment effectiveness, leaving open the possibility that unaccounted-for factors may influence the results.

One such factor could be differences in motivation and autonomy among patients across the referral pathways. According to SDT, internal motivation thrives when individuals experience autonomy, capability, and relatedness [32]. Self-referred patients may feel a greater sense of empowerment and autonomy by actively choosing to seek help through guided ICBT [31], which could enhance their motivation to engage with treatment more effectively compared to GP-referred patients [11]. The hope of recovery and the desire to gain control over one’s life, identified as internal motivators in a study by Wilhelmsen et al [64], may be particularly strong among self-referred patients, who make the decision to undergo therapy independently. This increased autonomy may enhance their motivation to engage with treatment [32]. In turn, higher motivation may lead to more effective engagement with ICBT tasks, which could boost self-referees’ sense of competence [33].

Other potential factors that may influence differences in treatment outcomes include both GPs’ beliefs about treatment and patients’ own beliefs and expectations. The assumption that “gold standard” psychotherapeutic treatment requires long-term, weekly face-to-face sessions with a therapist to be effective may shape how both GPs and patients perceive ICBT [65]. Patients often rely on their GPs for guidance in navigating the health care system, including for treatment recommendations [66]. A recent systematic review found that GPs’ negative attitudes toward ICBT can transfer to patients and potentially undermine treatment outcomes [67]. GP-referred patients who expected or preferred face-to-face therapy may have engaged less with ICBT, influencing their outcomes in this study. In contrast, self-referred participants actively chose ICBT, suggesting they may have had greater knowledge, motivation, and readiness for this treatment. Accordingly, patients’ perceptions of ICBT’s usefulness—and potential lack of awareness of its effectiveness—may impact patients’ engagement and treatment outcomes [68].

Guided ICBT relies on the patient’s ability to actively engage with the treatment and implement changes into their everyday life. Since sustained behavior change is more effective when driven by autonomous motivation [69], fostering this motivation may enhance treatment engagement. Enhancing patients’ sense of competence and connection may may further improve their engagement with homework, compliance with exposure exercises, and relapse prevention. These factors are crucial to therapy effectiveness, but have also been identified as major challenges in ICBT [70]. However, because technology is central to ICBT, factors like low computer self-efficacy, lack of basic computer skills, or computer anxiety may undermine perceived competence and hinder engagement with treatment [71], although previous studies have found that patients with lower levels of computer anxiety tend to show greater interest in ICBT [72]. Although these factors were not explicitly analyzed in our study, they may help contextualize the significant differences in treatment outcomes we found between the referral pathways.

Another factor potentially related to increased effectiveness in self-referred patients is educational level, as people with higher education report greater acceptance of digital mental health interventions than those with lower education [68]. Thus, the difference in education levels between referral pathways may contribute to differences in treatment outcomes. More self-referred patients had higher education, such as university degrees, compared to those referred by GPs. This is a pattern commonly observed across ICBT clinics and studies [20,24,73,74]. A key challenge in this context has been demonstrating that ICBT can effectively support individuals with fewer resources and lower education levels [24].

As education may develop capacities on many levels including increased sense of personal control, mastery, and self-direction [29], education may enhance engagement with ICBT by promoting autonomy and self-determination, as outlined in SDT [32,75]. Additionally, the treatment format in our study relies on text-heavy modules that guide patients through psychoeducation, working with negative or automatic thoughts and exposure therapy, a format known as bibliotherapy [76]. Patients with higher education levels may find it easier to process and apply these materials effectively, potentially contributing to their greater improvements.

Beyond comprehension, education is also linked to digital literacy, health literacy, and problem-solving skills, which may further support active engagement with the treatment format [29,30]. However, a recent systematic review found inconsistent evidence regarding the relationship between higher education and treatment outcomes in guided internet-delivered therapy, including ICBT [40]. This suggests that, while education may facilitate certain aspects of engagement, other factors such as motivation and readiness for digital interventions may play an equally important role.

Exploring the difference in treatment effectiveness between depression, PD, and SAD revealed large effect sizes of guided ICBT from pretreatment to posttreatment and from pre-treatment to the 6-month follow-up, regardless of referral pathway. These findings align with the large effects reported in systematic reviews for depression [63,77], PD [78,79], and SAD [80]. Our results indicate statistically significant differences in effect sizes between referral pathways across the 3 diagnoses.

In our study, both referral pathways led to significant symptom reduction, consistent with findings from research comparing GP referral and self-referral in psychological care [43] and musculoskeletal care [39]. Considering self-referral in a broader context, the United Kingdom’s IAPT program was introduced in 2007 within primary care to expand access to treatment for common mental health problems. Self-referral within IAPT has improved access to care, particularly for individuals who might not seek help through traditional GP referrals [27]. However, concerns have been raised about equity, as factors such as education, digital literacy, and ethnicity influence who engages with the program [81]. Recent research suggests that digital solutions, such as artificial intelligence–driven self-referral chatbots, may help reduce these disparities by increasing referrals from underrepresented groups, including ethnic minorities and nonbinary individuals [82]. Building on the IAPT, the Norwegian Prompt Access to Mental Health Care program provides adults with quick, low-threshold access to evidence-based treatment for anxiety and depression through self-referral. Situated within primary care, the program offers both short-term face-to-face therapy and guided internet-delivered treatment [83]. A randomized controlled trial comparing these modalities found that the digital option improves accessibility, requires significantly less therapist time, and has the potential to reach a larger population [84]. Sweden permits self-referral directly to specialized mental health care. A study of young Swedish adults who self-referred to a specialized mental health clinic found no evidence of overutilization of specialized services. Additionally, most self-referrers had not previously sought professional help for their psychiatric symptoms. This suggests that self-referral lowers the threshold for accessing specialized care especially for those who for various reasons do not contact their GPs for mental health problems [85].

### Limitations

First, the attrition was high, particularly at the 6-month follow-up, potentially introducing bias into the results, as indicated by the results from the missing data sensitivity analyses. This may, in part, reflect the limitations imposed by conducting the study within routine care, where ethical approval from the Regional Ethical Committee restricted patient contact to what was considered natural for the treatment process, and offering incentives for survey completion was not permitted. Future studies conducted outside these constraints could explore strategies such as using multiple contact methods or offering incentives to improve follow-up rates. Second, since we did not assess patients’ motivation for treatment, we can only speculate whether underlying patient characteristics such as those outlined by SDT [32] contributed to variations in effectiveness across referral pathways. Third, as we were only able to assess primary outcome measures for the diagnoses and did not assess secondary outcomes such as comorbidity disorders or quality of life, we cannot document a broader impact of the results. Fourth, although all data in this study were collected through the same secure web platform, technological advancements since data collection began in 2014 have surpassed the platform’s capabilities. Although extracting user data from the platform’s backend to examine engagement patterns and completion speeds would be valuable, this is not supported by the platform.

### Implications

Overall, our results showed that both referral pathways led to significant symptom reduction in guided ICBT. However, for patients with SAD, treatment effectiveness did not differ between referral pathways. These results underscore the importance of maintaining both referral pathways, as they attract distinct patient populations and serve complementary roles in facilitating access to mental health care. Although self-referral may be a more effective route for some, GPs remain crucial as gatekeepers, gate-openers, and trusted guides in navigating the health care system for others. Therefore, raising awareness among GPs and other health care providers about the viability and effectiveness of guided ICBT is essential to ensuring more patients benefit from this evidence-based treatment.

Delayed help-seeking remains a major challenge in mental health care [2,82]; our finding that the patients had been experiencing symptoms for an average of 10 years underscores the urgent need to increase help-seeking behavior. Improving mental health literacy could help reduce stigma and encourage help-seeking behavior [86], while public information campaigns may further raise awareness of the benefits of guided ICBT and self-referral. Additionally, our results suggest that motivation plays a key role in treatment success. Addressing potential barriers such as lower motivation in GP-referred patients through targeted engagement strategies could help optimize outcomes.

Beyond increasing accessibility, self-referral offers an essential pathway for individuals who may not seek help through traditional means [27,82]. Technology-driven solutions, such as artificial intelligence–powered self-referral chatbots, could further enhance access by reducing stigma, guiding users to appropriate services, and providing personalized interactions that support motivation and engagement [82]. Notably, chatbots have been shown to reduce negative attitudes and stigma toward mental health, potentially improving help-seeking behaviors [87]. Taken together, these findings highlight the need for a multifaceted approach that ensures timely and equitable access to mental health care while also addressing patient engagement and systemwide awareness.

### Future Directions

Future research could focus on conducting cost-effectiveness analyses of different referral pathways to provide policymakers and health care providers with a systematic comparison for prioritizing resource allocation. Additionally, exploring qualitative aspects of patient experiences with referral pathways may offer a more comprehensive understanding of the factors influencing treatment outcomes. Together, these approaches could guide the development of more effective and accessible ICBT services. Further, investigating the influence of potential mediators and moderators could clarify the relationship between referral pathways and treatment outcomes. Adjusting for factors like baseline characteristics may provide valuable insights into the mechanisms underlying guided ICBT effectiveness.

### Conclusion

This study demonstrates that both GP referral and self-referral pathways to guided ICBT are effective for moderate depression, PD, and SAD when delivered in a specialized routine care clinic. Notably, self-referred patients experienced significantly greater treatment outcomes both from pretreatment to posttreatment and from pretreatment to 6-month follow-up compared to those referred by a GP. We suggest that this additional improvement may stem from differences in internal motivation, with self-referred patients being more motivated to engage with the treatment, leading to greater long-term benefits. Our results highlight the vital role of self-referral and patient autonomy in driving sustained progress, particularly for depression and PD. At the same time, the comparable outcomes for SAD suggest that referral pathways may be less influential for this condition. These findings underscore the need for a health care system that supports both referral pathways, ensuring timely and equitable access to evidence-based psychological treatment while also fostering patient engagement and long-term recovery. Further research is needed to examine the effect of self-referral to guided ICBT when the pathway is implemented on a larger scale.

## Supplementary material

10.2196/68165 Multimedia Appendix 1 Estimated outcome measures over time for general practitioner–referred and self-referred groups.

10.2196/68165 Multimedia Appendix 2 Observed outcome measures preintervention, postintervention, and at 6-month follow-up.

10.2196/68165 Multimedia Appendix 3 Missing data sensitivity analyses.

## Abbreviations

BSQ: Body Sensation Questionnaire
CBT: cognitive behavioral therapy
FIML: full-information maximum likelihood
GP: general practitioner
IAPT: Improving Access to Psychological Therapies
ICBT: internet-based cognitive behavioral therapy
LMM: linear mixed modeling
MADRS-S: Montgomery Åsberg Depression Rating Scale, Self-rating version
MI: multiple imputation
MINI: Mini-International Neuropsychiatric Interview
PD: panic disorder
RCI: reliable change index
SAD: social anxiety disorder
SDT: Self-Determination Theory
SPS: Social Phobia Scale
