# Supplementary material for: Effectiveness of General Practitioner Referral Versus Self-Referral Pathways to Guided Internet-Delivered Cognitive Behavioral Therapy for Depression, Panic Disorder, and Social Anxiety Disorder: Naturalistic Study
Source: JMIR Ment Health. 2025 Mar 25;12:e68165. doi: 10.2196/68165 (PMC11962571; doi:10.2196/68165)
Supplement: Multimedia Appendix 1 [file mental-v12-e68165-s001.docx]

Table 1. Estimated outcome measures over time for GP and Self-referred groups.

|  | Harmonized  outcome | |  | MADRS-S | |  | BSQ | |  | SPS | |
| --- | --- | --- | --- | --- | --- | --- | --- | --- | --- | --- | --- |
|  | *b* | *P* |  | *b* | *P* |  | *b* | *P* |  | *b* | *P* |
| Pre | 46.13 | <.001 |  | 23.82 | <.001 |  | 43.15 | <.001 |  | 40.12 | <.001 |
| M1 ^a^ | -1.26 | .183 |  | -3.16 | .003 |  | -0.46 | .596 |  | 0.33 | .739 |
| M2 | -4.48 | <.001 |  | -3.98 | <.001 |  | -2.61 | .004 |  | -2.64 | .012 |
| M3 | -7.90 | <.001 |  | -4.37 | <.001 |  | -7.23 | <.001 |  | -3.62 | .001 |
| M4 | -10.47 | <.001 |  | -3.32 | .007 |  | -9.74 | <.001 |  | -6.23 | <.001 |
| M5 | -13.20 | <.001 |  | -4.95 | <.001 |  | -11.04 | <.001 |  | -8.99 | <.001 |
| M6 | -15.12 | <.001 |  | -7.13 | <.001 |  | -12.26 | <.001 |  | -9.50 | <.001 |
| M7 | -16.16 | <.001 |  | -11.25 | .005 |  | -12.32 | <.001 |  | -10.66 | <.001 |
| M8 | -27.77 | <.001 |  |  |  |  | -13.40 | .035 |  | -27.84 | <.001 |
| Post | -15.67 | <.001 |  | -5.15 | <.001 |  | -12.57 | <.001 |  | -11.68 | <.001 |
| Follow-up | -19.63 | <.001 |  | -7.54 | <.001 |  | -15.21 | <.001 |  | -14.40 | <.001 |
| Group differences: |  |  |  |  |  |  |  |  |  |  |  |
| Self-referred ^b^ | -2.26 | .229 |  | 0.50 | .756 |  | -1.42 | .444 |  | -2.55 | .350 |
| Self-referred*M1 | -2.52 | .079 |  | -0.74 | .639 |  | -1.23 | .402 |  | -1.83 | .326 |
| Self-referred*M2 | -3.40 | .024 |  | -0.58 | .719 |  | -3.34 | .030 |  | -1.79 | .366 |
| Self-referred*M3 | -3.74 | .017 |  | -2.00 | .228 |  | -1.04 | .513 |  | -4.35 | .036 |
| Self-referred*M4 | -3.99 | .014 |  | -4.83 | .005 |  | -1.12 | .504 |  | -2.48 | .248 |
| Self-referred*M5 | -3.83 | .022 |  | -3.35 | .058 |  | -2.17 | .205 |  | -2.67 | .239 |
| Self-referred*M6 | -3.62 | .036 |  | -2.73 | .134 |  | -2.14 | .224 |  | -2.25 | .329 |
| Self-referred*M7 | -5.00 | .013 |  |  |  |  | -3.18 | .080 |  | -3.01 | .208 |
| Self-referred*M8 | -2.67 | .829 |  |  |  |  | -6.90 | .450 |  |  |  |
| Self-referred*Post | -5.85 | <.001 |  | -6.60 | <.001 |  | -3.00 | .065 |  | -2.75 | .178 |
| Self-referred*Follow-up | -5.72 | .002 |  | -4.80 | .018 |  | -4.62 | .016 |  | -2.93 | .222 |

^a^ M: Module ^b^ Reference group: GP-referred
Pre: Pre-treatment; Post: Post-treatment; Follow-up: 6-months follow-up
MADRS-S: Montgomery Åsberg Depression Rating Scale, Self-rating version, BSQ: Body Sensation Questionnaire, SPS: Social Phobia Scale
