# Supplementary material for: Effectiveness of General Practitioner Referral Versus Self-Referral Pathways to Guided Internet-Delivered Cognitive Behavioral Therapy for Depression, Panic Disorder, and Social Anxiety Disorder: Naturalistic Study
Source: JMIR Ment Health. 2025 Mar 25;12:e68165. doi: 10.2196/68165 (PMC11962571; doi:10.2196/68165)
Supplement: Multimedia Appendix 3 [file mental-v12-e68165-s003.docx]

Table 3. Missing data sensitivity analyses

| **MADRS-S** |  |
| --- | --- |
| FIML | MI – 50 imputed data sets – all predictors |
| MODEL RESULTS  Two-Tailed  Estimate S.E. Est./S.E. P-Value  I ON  SELF REF 0.502 1.593 0.315 0.752  M1 ON  SELF REF -0.735 1.542 -0.476 0.634  M2 ON  SELF REF -0.579 1.588 -0.365 0.715  M3 ON  SELF REF -2.000 1.636 -1.222 0.222  M4 ON  SELF REF -4.824 1.703 -2.832 0.005  M5 ON  SELF REF -3.343 1.732 -1.931 0.054  M6 ON  SELF REF -2.731 1.795 -1.521 0.128  M7 ON  SELF REF 0.000 0.000 999.000 999.000  PO ON  SELF REF -6.594 1.720 -3.833 0.000  FU ON  SELF REF -4.796 1.995 -2.404 0.016  Intercepts  I 23.824 1.097 21.712 0.000  M1 -3.163 1.044 -3.029 0.002  M2 -3.976 1.077 -3.693 0.000  M3 -4.369 1.110 -3.935 0.000  M4 -3.315 1.195 -2.773 0.006  M5 -4.947 1.236 -4.002 0.000  M6 -7.129 1.301 -5.480 0.000  M7 -11.251 3.901 -2.884 0.004  PO -5.149 1.182 -4.357 0.000  FU -7.540 1.410 -5.348 0.000  Residual Variances  MAD0 26.513 1.627 16.294 0.000 | MODEL RESULTS  Two-Tailed Rate of  Estimate S.E. Est./S.E. P-Value Missing  I ON  SELF REF 0.375 1.750 0.214 0.830 0.352  M1 ON  SELF REF -0.072 1.778 -0.041 0.968 0.381  M2 ON  SELF REF 0.527 1.661 0.318 0.751 0.289  M3 ON  SELF REF -1.029 1.797 -0.573 0.567 0.395  M4 ON  SELF REF -4.317 1.828 -2.362 0.018 0.415  M5 ON  SELF REF -2.711 1.835 -1.477 0.140 0.420  M6 ON  SELF REF -2.330 1.870 -1.246 0.213 0.442  M7 ON  SELF REF 0.000 0.000 999.000 999.000 0.000  PO ON  SELF REF -6.209 1.820 -3.411 0.001 0.410  FU ON  SELF REF -5.490 1.973 -2.783 0.005 0.500  Intercepts  I 23.840 1.162 20.521 0.000 0.177  M1 -3.383 1.167 -2.899 0.004 0.194  M2 -4.329 1.198 -3.614 0.000 0.236  M3 -4.478 1.297 -3.453 0.001 0.350  M4 -3.586 1.513 -2.370 0.018 0.525  M5 -5.082 1.356 -3.749 0.000 0.406  M6 -7.741 1.334 -5.803 0.000 0.386  M7 -13.958 2.464 -5.665 0.000 0.896  PO -5.255 1.273 -4.127 0.000 0.325  FU -6.554 1.729 -3.789 0.000 0.639  Residual Variances  MAD0 33.042 2.674 12.355 0.000 0.669 |

MADRS-S: Montgomery Åsberg Depression Rating Scale, Self-rating version.

FIML= full-information maximum likelihood, SELF REF= self-referred, MI=missing data, M1-M7=module 1-7, PO=post-treatment, FU=6-month follow-up

| **BSQ** |  |
| --- | --- |
| FIML | MI – 50 imputed data sets – all predictors |
| MODEL RESULTS  Two-Tailed  Estimate S.E. Est./S.E. P-Value  I ON  SELF REF -1.421 1.838 -0.773 0.440  M1 ON  SELF REF -1.228 1.447 -0.848 0.396  M2 ON  SELF REF -3.337 1.517 -2.201 0.028  M3 ON  SELF REF -1.043 1.578 -0.661 0.509  M4 ON  SELF REF -1.118 1.654 -0.676 0.499  M5 ON  SELF REF -2.170 1.693 -1.281 0.200  M6 ON  SELF REF -2.144 1.742 -1.231 0.218  M7 ON  SELF REF -3.175 1.792 -1.772 0.076  m8 ON  SELF REF -6.907 9.025 -0.765 0.444  PO ON  SELF REF -2.999 1.605 -1.868 0.062  FU ON  SELF REF -4.620 1.898 -2.434 0.015  Intercepts  I 43.153 1.065 40.505 0.000  M1 -0.461 0.860 -0.536 0.592  M2 -2.612 0.899 -2.906 0.004  M3 -7.231 0.965 -7.492 0.000  M4 -9.740 0.990 -9.840 0.000  M5 -11.038 1.055 -10.467 0.000  M6 -12.262 1.084 -11.311 0.000  S7 -12.320 1.108 -11.115 0.000  M8 -13.590 6.353 -2.139 0.032  PO -12.568 0.945 -13.304 0.000  FU -15.210 1.136 -13.394 0.000  Residual Variances  BSQ0 36.147 1.684 21.471 0.000 | MODEL RESULTS  Two-Tailed Rate of  Estimate S.E. Est./S.E. P-Value Missing  I ON  SELF REF -1.426 1.735 -0.822 0.411 0.017  M1 ON  SELF REF -1.051 1.585 -0.663 0.507 0.093  M2 ON  SELF REF -3.633 1.601 -2.270 0.023 0.112  M3 ON  SELF REF -1.346 1.711 -0.787 0.431 0.223  M4 ON  SELF REF -0.388 1.697 -0.229 0.819 0.210  M5 ON  SELF REF -3.493 1.728 -2.022 0.043 0.239  M6 ON  SELF REF -1.815 1.670 -1.087 0.277 0.185  M7 ON  SELF REF -2.956 1.707 -1.732 0.083 0.220  M8 ON  SELF REF 1.327 1.621 0.819 0.413 0.134  PO ON  SELF REF -3.659 1.721 -2.126 0.034 0.233  FU ON  SELF REF -7.655 1.789 -4.278 0.000 0.291  Intercepts  I 43.158 1.008 42.819 0.000 0.052  M1 -0.410 0.933 -0.440 0.660 0.149  M2 -2.587 0.979 -2.641 0.008 0.229  M3 -7.053 1.024 -6.888 0.000 0.295  M4 -9.725 1.083 -8.979 0.000 0.371  M5 -9.825 1.076 -9.132 0.000 0.363  M6 -12.335 0.993 -12.425 0.000 0.250  M7 -12.174 1.113 -10.936 0.000 0.406  M8 -24.505 1.341 -18.271 0.000 0.593  PO -12.904 1.057 -12.204 0.000 0.340  FU -14.206 1.137 -12.498 0.000 0.430  Residual Variances  BSQ0 43.001 2.259 19.032 0.000 0.584 |

BSQ: Body Sensation Questionnaire

FIML= full-information maximum likelihood, SELF REF= self-referred, MI=missing data, M1-M7=module 1-7, PO=post-treatment, FU=6-month follow-up

| **SPS** |  |
| --- | --- |
| FIML | MI – 50 imputed data sets – all predictors |
| MODEL RESULTS  Two-Tailed  Estimate S.E. Est./S.E. P-Value  I ON  SELF REF -2.550 2.706 -0.942 0.346  M1 ON  SELF REF -1.831 1.845 -0.992 0.321  M2 ON  SELF REF -1.788 1.956 -0.914 0.361  M3 ON  SELF REF -4.348 2.049 -2.122 0.034  M4 ON  SELF REF -2.479 2.120 -1.169 0.242  M5 ON  SELF REF -2.672 2.243 -1.191 0.233  M6 ON  SELF REF -2.247 2.276 -0.987 0.324  M7 ON  SELF REF -3.006 2.363 -1.272 0.203  PO ON  SELF REF -2.750 2.019 -1.362 0.173  FU ON  SELF REF -2.929 2.372 -1.235 0.217  Intercepts  I 40.121 1.421 28.232 0.000  M1 0.330 0.980 0.336 0.737  M2 -2.644 1.041 -2.539 0.011  M3 -3.617 1.106 -3.269 0.001  M4 -6.230 1.177 -5.295 0.000  M5 -8.993 1.261 -7.130 0.000  M6 -9.498 1.320 -7.196 0.000  M7 -10.657 1.354 -7.870 0.000  PO -11.684 1.091 -10.710 0.000  FU -14.402 1.450 -9.931 0.000  Residual Variances  SPS0 51.335 2.504 20.497 0.000 | MODEL RESULTS  Two-Tailed Rate of  Estimate S.E. Est./S.E. P-Value Missing  I ON  SELF REF -2.642 2.658 -0.994 0.320 0.047  M1 ON  SELF REF -1.894 1.873 -1.012 0.312 0.115  M2 ON  SELF REF -2.164 1.985 -1.090 0.276 0.214  M3 ON  SELF REF -4.612 2.052 -2.248 0.025 0.265  M4 ON  SELF REF -3.499 2.000 -1.750 0.080 0.226  M5 ON  SELF REF -3.669 2.157 -1.701 0.089 0.336  M6 ON  SELF REF -3.178 2.118 -1.501 0.133 0.311  M7 ON  SELF REF -3.060 2.174 -1.408 0.159 0.346  PO ON  SELF REF -2.819 2.114 -1.334 0.182 0.308  FU ON  SELF REF -3.254 2.311 -1.408 0.159 0.423  Intercepts  I 40.191 1.394 28.842 0.000 0.054  M1 0.644 1.020 0.631 0.528 0.187  M2 -2.587 1.059 -2.444 0.015 0.247  M3 -3.634 1.175 -3.094 0.002 0.390  M4 -5.838 1.134 -5.147 0.000 0.345  M5 -8.527 1.240 -6.874 0.000 0.454  M6 -8.022 1.158 -6.929 0.000 0.372  M7 -9.008 1.145 -7.864 0.000 0.358  PO -11.163 1.131 -9.866 0.000 0.342  FU -15.260 1.589 -9.605 0.000 0.671  Residual Variances  SPS0 57.555 3.527 16.319 0.000 0.689 |

SPS: Social Phobia Scale

FIML= full-information maximum likelihood, SELF REF= self-referred, MI=missing data, M1-M7=module 1-7, PO=post-treatment, FU=6-month follow-up
